# Ultrasonography and 3D-CT Follow-Up of Extrahepatic Portal Vein Aneurysm: A Case Report

**DOI:** 10.1155/2010/560495

**Published:** 2010-06-14

**Authors:** Norio Yukawa, Makoto Takahashi, Kazuyoshi Sasaki, Takuma Mori, Ayumi Matsuo, Kuniyasu Ito, Hiroyuki Kirikoshi, Kiyoshi Ohya, Nobuyuki Wada, Yasushi Rino, Munetaka Masuda

**Affiliations:** ^1^Kamishirane Hospital, 2-65-1 Kamishirane, Asahi-ku, Yokohama 241-0002, Japan; ^2^Department of Surgery, Yokohama City University, 3-9 Fukuura, Kanazawa-ku, Yokohama 236-0004, Japan; ^3^Department of Gastroenterology, Yokohama City University, Yokohama 236-0004, Japan

## Abstract

Extrahepatic portal vein aneurysm is a rare disorder. From 1956 to 2008, we found only 43 published English-language reports, including 67 cases, using Pub Med. We report a case of a 77-year-old woman who had complaints of lower abdominal fullness and residual urine. We performed ultrasonography (US), which demonstrated a congenital extrahepatic portal vein aneurysm. She had no obvious symptoms of the extrahepatic portal vein aneurysm. She had undergone gastrectomy without blood transfusion for gastric ulcer more than 20 years ago. Physical examination revealed no abnormal findings. US revealed a 2.2 × 1.8 cm, round shaped hypoechogenic lesion at the hepatic hilum. Color Doppler US showed bidirectional colors due to circular flow within this lesion. 3D-CT and CT angiography demonstrated that the saccular aneurysm at the hepatic hilum was 3.0 cm in diameter and was enhanced equal to that of portal vein.Twenty-six months after the diagnosis, the aneurysm had not grown in size. Since our patient had no serious complaints or liver disease, surgical procedures had not been employed. US and 3D-CT are noninvasive diagnostic techniques and are helpful in the diagnosis and follow-up of extrahepatic portal vein aneurysms.

## 1. Case report

**Figure 1:**
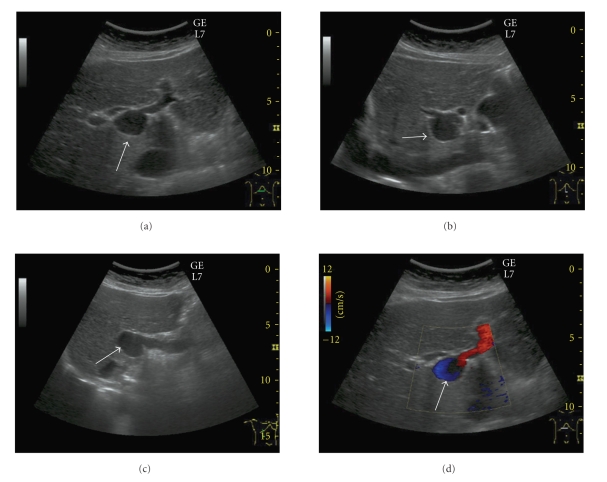
(a), (b), and (c) Ultrasonography (US) revealed a well-circumscribed anechoic structure that was 26.4 × 21.7 × 19.4 mm in size at the porta hepatis (white arrow). (d) Color Doppler US demonstrated bidirectional color due to circular flow within the aneurysm.

**Figure 2:**
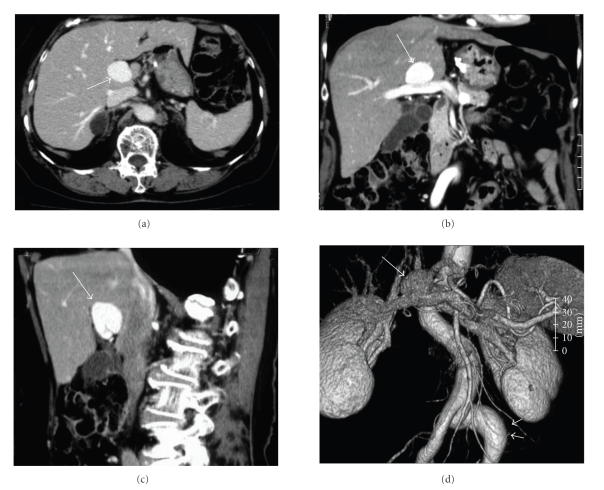
3D-CT (a) horizontal view, (b) coronary view, and (c): sagittal view and CT angiography (d) demonstrated that the saccular aneurysm at the hepatic hilum was 30 mm in diameter and was enhanced equal to that of the portal vein (white arrow). CT angiography demonstrated left common iliac artery aneurysm (white arrow head).

A 77-year-old Japanese woman had been hospitalized in our institute due to urocystitis. In September 2007, she was incidentally diagnosed with a congenital extrahepatic portal vein aneurysm after Ultrasonography (US) was conducted during an investigation for lower abdominal fullness and residual urine. She had undergone gastrectomy due to gastric ulcer without blood transfusion. Her family history was not particularly interesting. Physical examination revealed no jaundice, no abdominal mass, and no vascular noise. Routine blood tests did not show any remarkable findings. US revealed a well-circumscribed anechoic structure that was 26.4 × 21.7 × 19.4 mm in size at the porta hepatis (Figures 1(a), 1(b), and 1(c)). Color Doppler US demonstrated bidirectional color due to circular flow within the aneurysm (Figure 1(d)). Abdominal enhanced 3D-CT and CT angiography demonstrated that the saccular aneurysm at the hepatic hilum was 30 mm in diameter and was enhanced equal to that of portal vein (Figures 2(a), 2(b), and 2(c)). CT angiography demonstrated a common iliac artery aneurysm (Figure 2(d)).

She remained asymptomatic and returned to the hospital 26 months after the diagnosis for follow-up. The aneurysm showed no enlargement in size by follow-up CT and US after 27 months.

## 2. Discussion

Portal vein aneurysm (PVA) is an uncommon disease [1, 2]. However, recently, the number of reported cases has gradually increased with advances in imaging techniques. Koc et al. [3] reported 25 PVA cases in 4186 patients using abdominal CT and US. PVAs are classified as intrahepatic and extrahepatic. Our case was diagnosed as extrahepatic PVA. Sixty-seven cases of extrahepatic PVAs were reported in the English literature from 1956 to 2008 [1–44]. Including our case, we summarized 68 PVA cases (Table 1). Twenty-three males, 44 females, and one unknown were reported. Their mean age was 51.4 years (range 0–83 years), and the mean size was 43.6 mm (range 8–115 mm). The cyst-form was found in 36 cases and the fusiform was found in 22 cases (unknown 11 cases). Half of all cases complained of abdominal or back pain. Nineteen cases had no symptoms. Twenty-one patients showed liver dysfunction in blood examination, and only 23 had portal hypertension. Half of all cases had no past history. Nineteen patients had undergone operation, 2 had undergone embolization by angiography, and 46 were observed clinically. Fifty-eight cases had a good course, while 7 were dead. Previously, most patients with asymptomatic PVAs were managed by observation [44]. Recently, several reports have suggested that operation can be performed easily and safely [1, 44]. The operation may be one of the therapeuticprocedures for PVAs with any symptoms or complications. 

The origin of PVAs may be congenital or acquired [37]. Formerly, cases of PVA with liver disorder, portal hypertension, trauma, or pancreatitis were reported, but recently congenital PVA cases have been reported without these diseases, due to advances in imaging [1, 6]. Our case was considered to be congenital PVA due to the absence of past history and the presence of common iliac artery aneurysm. 

Abdominal US (including Doppler US) is useful and convenient for the diagnosis of PVA and has been used in most cases to date [8, 9, 34]. About 70% of reported extra PVAs had undergone US. By contrast, angiography can describe PVA collectively and clearly [31, 32] but is not indispensable because of its invasive nature. In place of angiography, MRI (including MR-angio) has been used increasingly [5]. Our patient had undergone gastrectomy twenty years ago and stainless staples remained in the abdomen. Because of those staples, 3D-CT was selected to describe a solid image of the whole portal vein system. 3D-CT can show the size and location of PVA in stereoimages. Repeatability is high without interoperator variability and may confirm the diagnosis and the observation of PVA. 

## Figures and Tables

**Table 1:** Summary of the reported cases of portal vein aneurysm in English litelature (including our case).

Sex				Liver disorder	
Male		23		positive	21
Female		44	Total 68	negative	45
unknown		1		unknown	2

Age	51.4 (0~83)			Portal hypertension	
				positive	23
				negative	45

Size			Size (cm)	Congenital or acquired	
Extrahepatic	67		average	congenital	39
Extra-Intrahepatic	1		4.36 (0.8~11.5)	acquired	5
				unknown	24

Shape					
cyst-form	36		4.20 (0.8~11.5)		
fusiform	22		4.62 (0.8~8.0)		
unknown	11				

Symptoms				Past history	
Abdominal or back pain	34			hepatitis, liver cirrhosis, HCC	12
nausea, vomiting	6			esophageal varix	4
hematemesis, melena	3			gallstone, cholecystectomy	5
fever up	4			hysterectomy	2
jaundice	4			Malignancy (exclude HCC)	3
liver disorder	1			gastric ulcer	2
indigestion	1			gastrectomy	2
rapture	1			pancreatitis	1
hematuria	1			arthritis	1
abdominal discomfort	1			hypertension	2
splenomegaly	1			diabetes mellitus	1
chorecystitis	1			idiopathic portal hypertension	1
none	19			hyperammoninemia	1
unknown	1			lower extremity varix	1
	*including overlap			non-chirrhotic portal fibrosis	1
				schistosomiasis	1
				myocardial infarction	1
				SLE	1
				none	34
				unknown	1
					*including overlap

Tool of diagnosis				Treatment	
ultrasonography	48			none	46
CT	43			operation	19
angiography	21			Angiography, embolization	2
MRI	14			unknown	1
scintigram	2			Clinical course	
none	2			good course	58
autopsy	1			dead	7
unknown	1			unknown	3
	*including overlap				
